# Supplementary material for: MicroRNA expression profiles in molecular subtypes of clear-cell renal cell carcinoma are associated with clinical outcome and repression of specific mRNA targets
Source: PLoS One. 2020 Sep 11;15(9):e0238809. doi: 10.1371/journal.pone.0238809 (PMC7485767; doi:10.1371/journal.pone.0238809)
Supplement: S2 Table — All miRs except for miR 30c-5p remained independent predictors for OS. Molecular subtype remained and independent predictor as well, except when tested against miRs 21-3p (up in unfavorable ccrcc1_4) and 204-5p (up in favorable ccrcc2_3). IMDC risk groups: good (G), intermediate (I) and poor (P). (DOCX) [file pone.0238809.s002.docx]

| **miRNA and IMDC** | | | **miRNA and molecular subtype** | | | |
| --- | --- | --- | --- | --- | --- | --- |
|  | **p-value** | **hazard ratio** |  | **p-value** | | **hazard ratio** |
| **let-7i-5p** | 0,0001 | 1,7 | **let-7i-5p** | 0,003 | | 1,6 |
| **IMDC-I vs G** | 0,082 | 1,9 | **ccrcc2_3 vs 1_4** | 0,033 | | 0,6 |
| **IMDC-P vs G** | 0,001 | 3,7 |  |  | |  |
| **21-3p** | 0,0001 | 1,4 | **21-3p** | 0,006 | | 1,4 |
| **IMDC-I vs G** | 0,068 | 2,0 | **ccrcc2_3 vs 1_4** | 0,057 | | 0,6 |
| **IMDC-P vs G** | 0,005 | 3,2 |  |  | |  |
| **21-3p** | < 0,0001 | 1,7 | **21-3p** | 0,003 | | 1,6 |
| **IMDC-I vs G** | 0,034 | 2,2 | **ccrcc2_3 vs 1_4** | 0,010 | | 0,5 |
| **IMDC-P vs G** | 0,0001 | 4,1 |  |  | |  |
| **30c-5p** | 0,095 | 0,9 | **30c-5p** | 0,124 | | 0,8 |
| **IMDC-I vs G** | 0,058 | 2,0 | **ccrcc2_3 vs 1_4** | 0,019 | | 0,5 |
| **IMDC-P vs G** | 0,002 | 3,6 |  |  | |  |
| **34c-5p** | < 0,0001 | 1,3 | **34c-5p** | 0,004 | | 1,2 |
| **IMDC-I vs G** | 0,036 | 2,2 | **ccrcc2_3 vs 1_4** | 0,020 | | 0,6 |
| **IMDC-P vs G** | 0,002 | 3,5 |  |  | |  |
| **134-5p** | 0,007 | 1,2 | **134-5p** | 0,034 | | 1,2 |
| **IMDC-I vs G** | 0,049 | 2,1 | **ccrcc2_3 vs 1_4** | 0,021 | | 0,6 |
| **IMDC-P vs G** | 0,001 | 3,6 |  |  | |  |
| **135a-5p** | 0,001 | 0,8 | **135a-5p** | 0,038 | | 0,9 |
| **IMDC-I vs G** | 0,024 | 2,3 | **ccrcc2_3 vs 1_4** | 0,013 | | 0,5 |
| **IMDC-P vs G** | 0,001 | 4,0 |  |  | |  |
| **146b-5p** | < 0,0001 | 1,4 | **146b-5p** | 0,001 | | 1,4 |
| **IMDC-I vs G** | 0,036 | 2,2 | **ccrcc2_3 vs 1_4** | 0,008 | | 0,5 |
| **IMDC-P vs G** | 0,002 | 3,5 |  |  | |  |
| **193b-5p** | 0,002 | 1,2 | **193b-5p** | 0,036 | | 1,2 |
| **IMDC-I vs G** | 0,031 | 2,3 | **ccrcc2_3 vs 1_4** | 0,008 | | 0,5 |
| **IMDC-P vs G** | 0,001 | 3,7 |  |  | |  |
| **204-5p** | < 0,0001 | 0,7 | **204-5p** | < 0,0001 | | 0,7 |
| **IMDC-I vs G** | 0,045 | 2,1 | **ccrcc2_3 vs 1_4** | 0,109 | | 0,7 |
| **IMDC-P vs G** | 0,003 | 3,3 |  |  | |  |
| **425-5p** | 0,003 | 1,6 | **425-5p** | 0,032 | | 1,5 |
| **IMDC-I vs G** | 0,016 | 2,5 | **ccrcc2_3 vs 1_4** | 0,011 | | 0,5 |
| **IMDC-P vs G** | 0,001 | 3,7 |  |  | |  |
| **1260a** | < 0,0001 | 0,7 | **1260a** | 0,003 | | 0,8 |
| **IMDC-I vs G** | 0,037 | 2,2 | **ccrcc2_3 vs 1_4** | 0,011 | | 0,5 |
| **IMDC-P vs G** | 0,001 | 3,7 |  |  | |  |
| **1307-3p** | 0,010 | 1,3 | **1307-3p** | 0,017 | | 1,3 |
| **IMDC-I vs G** | 0,062 | 2,0 | **ccrcc2_3 vs 1_4** | 0,004 | | 0,5 |
| **IMDC-P vs G** | 0,001 | 3,7 |  |  | |  |
| **7977** | < 0,0001 | 0,7 | **7977** | < 0,0001 | | 0,7 |
| **IMDC-I vs G** | 0,048 | 2,1 | **ccrcc2_3 vs 1_4** | 0,008 | | 0,5 |
| **IMDC-P vs G** | 0,001 | 3,9 |  |  |  | |
