# Supplementary material for: MicroRNA expression profiles in molecular subtypes of clear-cell renal cell carcinoma are associated with clinical outcome and repression of specific mRNA targets
Source: PLoS One. 2020 Sep 11;15(9):e0238809. doi: 10.1371/journal.pone.0238809 (PMC7485767; doi:10.1371/journal.pone.0238809)
Supplement: S1 Fig — Clustering reveals two miRNA clusters with 66% and 91% overlap with favorable ccrcc2_3 and unfavorable ccrcc1_4 molecular subtypes respectively (p = 4.7e-9). (PDF) [file pone.0238809.s003.pdf]

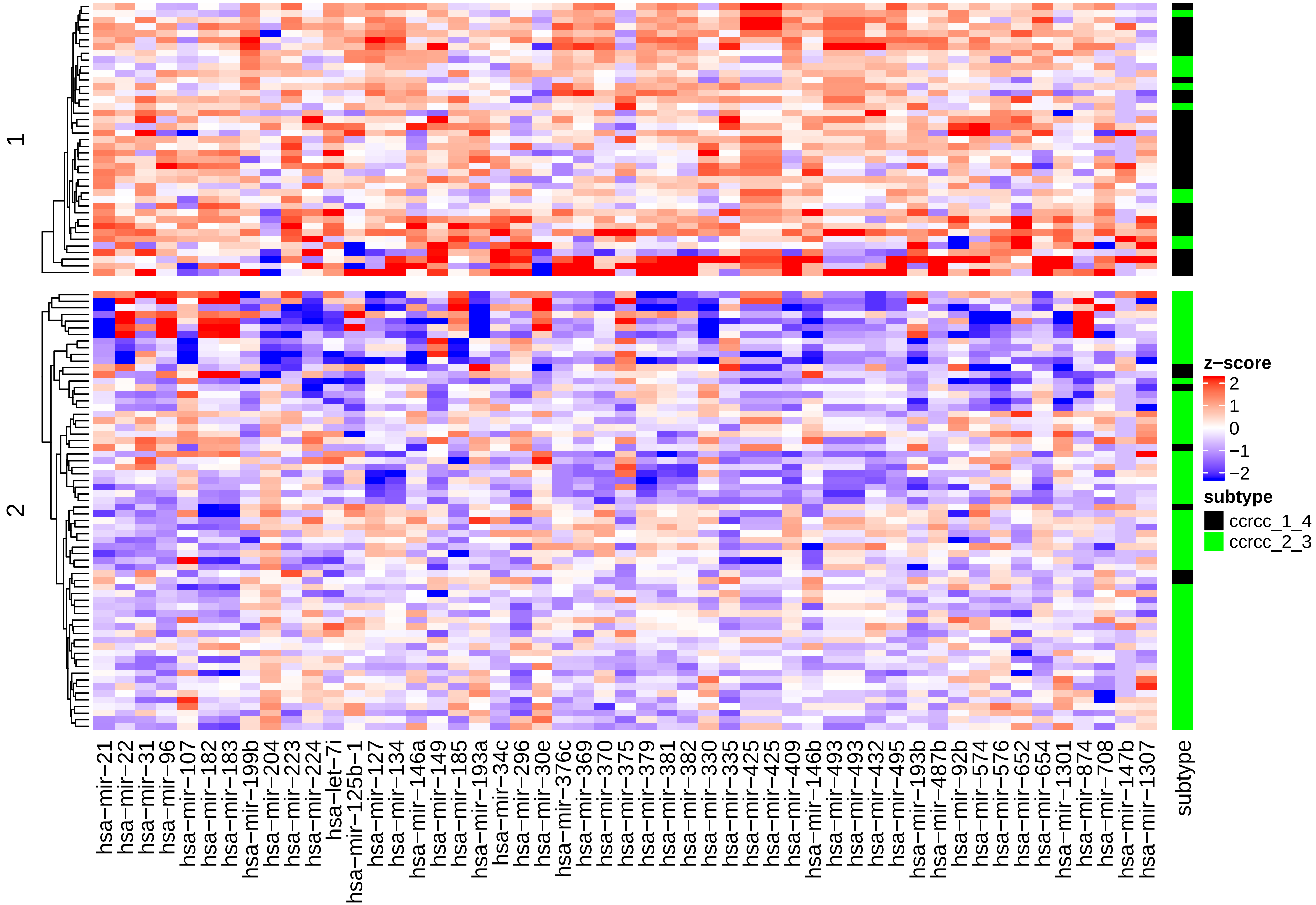

1
2
z−score
2
1
0
−1
−2
subtype
ccrcc_1_4
ccrcc_2_3
hsa−mir−21
hsa−mir−22
hsa−mir−31
hsa−mir−96
hsa−mir−107
hsa−mir−182
hsa−mir−183
hsa−mir−199b
hsa−mir−204
hsa−mir−223
hsa−mir−224
hsa−let−7i
hsa−mir−125b−1
hsa−mir−127
hsa−mir−134
hsa−mir−146a
hsa−mir−149
hsa−mir−185
hsa−mir−193a
hsa−mir−34c
hsa−mir−296
hsa−mir−30e
hsa−mir−376c
hsa−mir−369
hsa−mir−370
hsa−mir−375
hsa−mir−379
hsa−mir−381
hsa−mir−382
hsa−mir−330
hsa−mir−335
hsa−mir−425
hsa−mir−425
hsa−mir−409
hsa−mir−146b
hsa−mir−493
hsa−mir−493
hsa−mir−432
hsa−mir−495
hsa−mir−193b
hsa−mir−487b
hsa−mir−92b
hsa−mir−574
hsa−mir−576
hsa−mir−652
hsa−mir−654
hsa−mir−1301
hsa−mir−874
hsa−mir−708
hsa−mir−147b
hsa−mir−1307
subtype
