# Supplementary figures and images for: MicroRNA expression profiles in molecular subtypes of clear-cell renal cell carcinoma are associated with clinical outcome and repression of specific mRNA targets
Source: PLoS One. 2020 Sep 11;15(9):e0238809. doi: 10.1371/journal.pone.0238809 (PMC7485767; doi:10.1371/journal.pone.0238809)

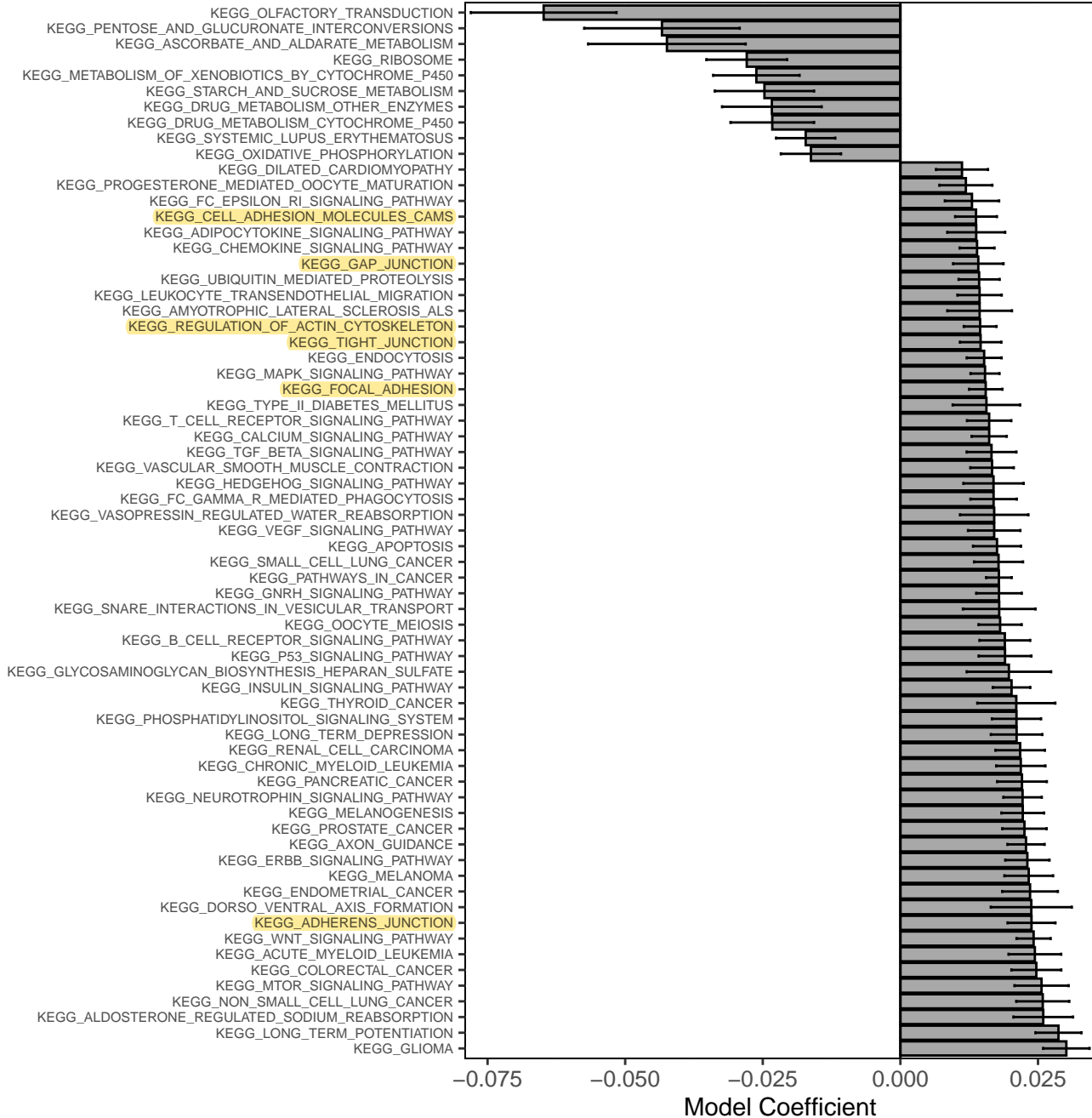

Supplement: S2 Fig — KEGG analysis showed less suppression of pathways involved in retaining an epithelial phenotype in ccrcc2_3 tumors, suggesting less possibility for epithelial-to-mesenchymal transition in these tumors. The biological interpretation of the model coefficient can be stated as follows (in the context of two-group comparison, i.e., ccrcc2_3 vs ccrcc1_4): if the coefficient is positive, miRNA inhibition on target mRNAs might be lifted, thereby leading to less suppression on the gene set of interest in the experimental group. (PDF) [file pone.0238809.s004.pdf]

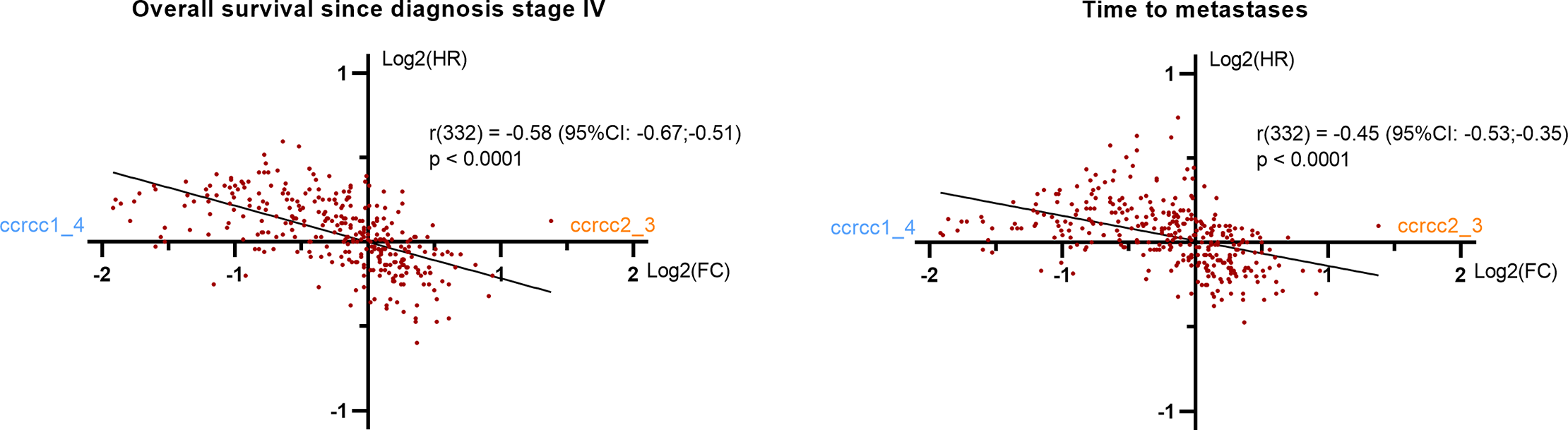

Supplement: S3 Fig — Differences in miRNA expression were similar across outcome parameters: OS since diagnosis (main text), OS since stage IV (A) and time to metastases (B). HR = hazard ratio; FC = fold change. (TIF) [file pone.0238809.s005.tif]
