# Supplementary material for: MicroRNA expression profiles in molecular subtypes of clear-cell renal cell carcinoma are associated with clinical outcome and repression of specific mRNA targets
Source: PLoS One. 2020 Sep 11;15(9):e0238809. doi: 10.1371/journal.pone.0238809 (PMC7485767; doi:10.1371/journal.pone.0238809)
Supplement: Attachment — Submitted filename: Response to reviewers.docx [file pone.0238809.s006.docx]

Reviewer #1: The question, study execution, and results are straightforward. Also, few technical issues are either not discussed or not addressed. The authors must carefully address all of my concerns before further consideration.

*We thank the reviewer for his/her appreciation of the manuscript.*

1. The figure quality should be improved.

*The figure quality is indeed greatly reduced in the pdf file for peer review. The separate figure files are high quality and adhere to Plos One requirements, including a quality check by PACE.*

2. Line number: 59
Please cite reference in proper place.

*The authors are not entirely sure what is meant by this comment? The reference is cited between brackets at the end of the phrase where it is first mentioned (we have opted to systematically cite at the end of sentences and not in the middle).*

3. Line number: 116
Please provide the corresponding figure validated by tcga dataset.

*We have included it as Supporting Figure 1.*

4. Line number: 186
What is the full form of OS?

*We thank the reviewer for noticing that the abbreviation was incorrectly explained only in figure/table legends. We added “overall survival (OS)” at its first appearance in the text.*

5. Line number: 215
Please provide a figure/ table to support “MiRNA expression is not predictive for response to sunitinib or pazopanib”

*The results have been provided in Supporting Table 1.*
